# Prognostic value of preoperative inflammatory response biomarkers in patients with esophageal cancer who undergo a curative thoracoscopic esophagectomy

**DOI:** 10.1186/s12893-016-0179-5

**Published:** 2016-09-20

**Authors:** Noriyuki Hirahara, Takeshi Matsubara, Yoko Mizota, Shuichi Ishibashi, Yoshitsugu Tajima

**Affiliations:** Department of Digestive and General Surgery, Shimane University Faculty of Medicine, 89-1 Enya-cho, Izumo, Shimane 693-8501 Japan

**Keywords:** Esophageal cancer, Lymphocyte to monocyte ratio (LMR), Neutrophil to lymphocyte ratio (NLR), Platelet lymphocyte ratio (PLR), Prognostic predictor

## Abstract

**Background:**

Several inflammatory response biomarkers, including lymphocyte-to-monocyte ratio (LMR), neutrophil-to-lymphocyte ratio (NLR), and platelet-to-lymphocyte ratio (PLR) have been reported to predict survival in various cancers. The aim of this study is to evaluate the clinical value of these biomarkers in patients undergoing curative resection for esophageal cancer.

**Methods:**

The LMR, NLR and PLR were calculated in 147 consecutive patients who underwent esophagectomy between January 2006 and February 2015. We examined the prognostic significance of the LMR, NLR, and PLR in both elderly and non-elderly patients. We evaluated the cancer-specific survival (CSS), with the cause of death determined from the case notes or computerized records.

**Results:**

Univariate analyses demonstrated that TNM pStage (*p* < 0.0001), tumor size (*p* = 0.0014), operation time (*p* = 0.0209), low LMR (*p* = 0.0008), and high PLR (*p* = 0.0232) were significant risk factors for poor prognosis. Meanwhile, TNM pStage (*p* < 0.0001) and low LMR (*p* = 0.0129) were found to be independently associated with poor prognosis via multivariate analysis.

In non-elderly patients, univariate analyses demonstrated that TNM pStage (*p* < 0.0001), tumor size (*p* = 0.0001), operation time (*p* = 0.0374), LMR (*p* < 0.0001), and PLR (*p* = 0.0189) were significantly associated with a poorer prognosis. Multivariate analysis demonstrated that TNM pStage (*p* = 0.001) and LMR (*p* = 0.0007) were independent risk factors for a poorer prognosis.

In elderly patients, univariate analysis demonstrated that that TNM pStage (*p* = 0.0023) was the only significant risk factor for a poor prognosis.

**Conclusions:**

LMR was associated with cancer-specific survival (CSS) of esophageal cancer patients after curative esophagectomy. In particular, a low LMR was a significant and independent predictor of poor survival in non-elderly patients. The LMR was convenient, cost effective, and readily available, and could thus act as markers of survival in esophageal cancer.

## Background

It is now widely recognized that host-related factors, such as performance status, weight loss, smoking, and comorbidity, as well as the biological properties of individual tumors, play an important role in cancer outcomes [1]. Recent studies have shown that preoperative inflammation-based prognostic scores have a significant predictive and prognostic value in various types of cancers [2–4]. A systemic inflammatory response has been reported to be associated with tumor development, apoptosis inhibition, and angiogenesis promotion, thus resulting in tumor progression and metastasis [5, 6]. Furthermore, significant relationships between patient survival and the lymphocyte-to-monocyte ratio (LMR), neutrophil-to-lymphocyte ratio (NLR), and platelet-to-lymphocyte ratio (PLR) have been documented in various cancers [7–9]. However, only a few studies have evaluated the utility of inflammation-based scores for assessing the prognosis of patients with esophageal cancer.

The aim of the present study was to evaluate whether the LMR, NLR, and PLR have prognostic values independent of conventional clinicopathological features in patients undergoing a potentially curative resection for esophageal cancer. Additionally, this study stratified patients into two age groups, elderly patients aged 70 years or older and patients aged under 70 years, because esophageal cancer occurs predominantly in elderly people and age-specific prognostic factors in patients with esophageal cancer have not yet been identified.

## Methods

### Patients

We retrospectively reviewed a database of medical records from 147 consecutive patients who underwent curative esophagectomy with R0 resection for histologically verified esophageal squamous cell carcinoma between January 2006 and February 2015 at Shimane University Faculty of Medicine. R0 resection was defined as a complete resection without any microscopic resection margin involvement. Video-assisted or thoracoscopic subtotal esophagectomy with three-field lymph node dissection was performed in all patients, followed by laparoscopic gastric surgery with an elevation of the gastric conduit to the neck via the posterior mediastinal or a retrosternal approach with an end-to-end anastomosis of the remnant cervical esophagus and fundus of the gastric conduit. The patients’ clinical characteristics, laboratory data, treatment, and pathological data were obtained from medical records. Preoperatively, no patients had clinical signs of infection or other systemic inflammatory conditions. Based on the age distribution of the patients, they were subdivided into two groups in this study: patients <70 years (non-elderly group) and patients ≥70 years (elderly group). We evaluated cancer-specific survival (CSS), with the cause of death determined from case notes or computerized records.

This retrospective study was approved with the ethical board of Shimane University Faculty of Medicine, and was conducted in accordance with the Declaration of Helsinki. Informed consent was obtained from all individual participants included in the study.

### Blood sample analysis

Data on preoperative complete blood cell (CBC) counts were retrospectively extracted from patient medical records. Only patients with available preoperative CBC count and blood differential data were included in the study. All white blood cell and differential counts were obtained within 1 week prior to surgery. CBC was measured using ethylenediaminetetraacetic acid-treated blood, and analyzed using an automated hematology analyzer XE-5000 (SYSMEX K1000 hematology analyzer; Medical Electronics, Kobe, Japan). Absolute counts of lymphocytes, monocytes, and platelets were obtained from CBC tests.

### LMR, NLR, and PLR evaluations

The LMR was calculated from a routinely performed preoperative blood cell count as the absolute lymphocyte count divided by the absolute monocyte count. White blood cell count data were analyzed in the general routine laboratory of our hospital. The NLR was calculated as a simple ratio between the absolute neutrophil and absolute lymphocyte counts, as provided by the differential white blood cell count. The PLR was calculated from the differential count by dividing the absolute platelet count by the absolute lymphocyte count.

### TNM stage

The pathological classification of the primary tumor, degree of lymph node involvement, and presence of organ metastasis were determined according to the TNM classification system [10].

### Statistical analysis

Means and standard deviations were calculated, and differences between groups were evaluated using a Student’s *t*-test. Differences between categories of each clinicopathological feature were analyzed using a Chi-square (*χ*^2^) test.

We determined the optimal cut-off levels of the LMR, NLR, and PLR by applying receiver operating curve (ROC) analysis. Regarding LMR, the area under curve (AUC) was 0.69 for CSS. A value of 4.0 was chosen as the cut-off level for LMR for CSS as associated with a high sensitivity and specificity for CSS (62.5 and 71.3 %, respectively). Regarding NLR, the AUC was 0.58 for CSS. A value of 1.6 was chosen as the cut-off level for NLR for CSS as associated with a sensitivity and specificity for CSS (57.5 and 66.3 %, respectively). Regarding PLR, the AUC was 0.65 for CSS. A value of 147 was chosen as the cut-off level for PLR for CSS as associated with a high sensitivity and specificity for CSS (59.6 and 68.4 %, respectively). The patients with LMR, NLR, and PLR greater than these cutoff values were considered to have high LMR, NLR, and PLR, respectively; the remaining patients were considered to have low LMR, low NLR, and low PLR. CSS was calculated using Kaplan–Meier analysis, and differences between the groups were assessed by a log-rank test. Additionally, prognostic factors associated with decreased survival rates were determined using Cox regression analysis.

Univariate analyses were performed to determine which variables were associated with CSS. Variables with a *p*-value <0.05 in univariate analysis were subjected to multivariate logistic regression analysis. The potential prognostic factors for esophageal cancer were as follows: age (<70 vs. ≥70 years); sex (female vs. male); pStage (I, II vs. III); tumor size (<3 cm vs. ≥3 cm); operation time (<600 vs. ≥600 min); intraoperative blood loss (<5 00 mL vs. ≥500 mL); LMR (≥4 vs. <4); NLR (≥1.6 vs. <1.6); PLR (<147 vs. ≥147); weight loss (No vs. Yes: Weight loss was defined as more than 5 % decreasing in the body weight in the last 3 months preceding operation); and serum squamous cell carcinoma (SCC) antigen value (<1.5 vs. ≥1.5). Medical records were retrospectively reviewed to examine these factors.

All statistical analyses were performed using the statistical software JMP (version 11 for Windows; SAS Institute, Cary, NC, USA), and *p*-values <0.05 were considered statistically significant.

## Results

### Relationships between LMR, NLR, PLR, and clinicopathological features in patients with esophageal cancer

The relationships between LMR, NLR, PLR, and clinicopathological features in 147 patients with esophageal cancer are shown in Table 1.

**Table 1:** Relationships between LMR, NLR, PLR and clinicopathologic features of 147 all patients

Characteristics	Total patients	LMR			NLR			PLR		
		<4 (*n* = 64)	≥4 (*n* = 83)	*p* value	1.6< (*n* = 37)	≥1.6 (*n* = 110)	*p* value	147< (*n* = 79)	≥147 (*n* = 68)	*p* value
Age (years)		65.8 ± 7.4	65.7 ± 8.2	0.934	65.4 ± 8.0	65.9 ± 7.9	0.72	66.8 ± 8.1	64.6 ± 7.6	0.097
Gender				0.052			0.163			0.562
Male	132	61	71		31	101		72	60	
Female	15	3	12		6	9		7	8	
WBC		6082.2 ± 2153.2	5844.3 ± 1788.2	0.466	5284.1 ± 1667.3	6171.2 ± 1996.5	0.016	6190.9 ± 1723.0	5665.6 ± 2167.2	0.104
Neutrophil		3944.7 ± 1804.6	3412.8 ± 1470.4	0.051	2491.0 ± 948.3	4032.3 ± 1643.7	<0.0001	3509.3 ± 1300.5	3801.3 ± 1960.9	0.283
Lymphocyte		1322.0 ± 546.4	1942.5 ± 584.5	<0.0001	2187.6 ± 658.6	1499.0 ± 541.8	<0.0001	2029.2 ± 586.3	1257.7 ± 426.2	<0.0001
Monocyte		546.8 ± 211.3	328.7 ± 111.1	<0.0001	379.0 ± 161.3	438.7 ± 203.3	0.1074	418.2 ± 171.3	430.0 ± 220.2	0.714
Platelet		236.6 ± 79.2	226.9 ± 66.2	0.42	231.0 ± 76.9	231.2 ± 70.7	0.987	203.5 ± 49.2	263.2 ± 80.9	<0.0001
Location of tumor				0.09			0.313			0.042
Ce	6	5	1		1	5		0	6	
Ut	8	4	4		0	8		5	3	
Mt	65	29	36		20	45		32	33	
Lt	52	23	29		11	41		31	21	
Ae	16	3	13		5	11		11	5	
Tumor size (mm)		4.9 ± 1.9	3.9 ± 2.7	0.014	3.8 ± 2.8	4.5 ± 2.3	0.134	4.0 ± 2.5	4.8 ± 2.3	0.056
Depth of tumor				0.0007			0.002			0.06
T1a-1b	66	20	46		18	48		40	26	
2	12	2	10		8	4		9	3	
3	56	33	23		8	48		26	30	
4a-4b	13	9	4		3	10		4	9	
Lymph node metastasis				0.2732			0.1532			0.0639
N0	79	30	49		22	57		43	36	
N1	42	19	23		12	30		25	17	
N2	12	8	4		3	9		8	4	
N3	14	7	7		0	14		3	11	
Pathological stage				0.0002			0.1338			0.3497
1a-1b	59	14	45		20	39		36	23	
2a-2b	33	21	12		6	27		16	17	
3a-3c	55	29	26		11	44		27	28	
Operation time (min)		644.8 ± 162.2	663.5 ± 159.2	0.4843	655.9 ± 177.2	655.2 ± 155.0	0.9798	676.5 ± 149.0	630.8 ± 170.2	0.0845
Intraoperative blood loss (ml)		751.8 ± 622.8	581.6 ± 633.4	0.1059	568.8 ± 511.1	684.9 ± 667.8	0.3359	598.5 ± 633.1	722.2 ± 629.7	0.2384
SCC antigen		1.19 ± 1.06	1.12 ± 1.12	0.7208	1.04 ± 1.12	1.19 ± 1.08	0.7643	1.05 ± 0.91	1.27 ± 1.26	0.8858

*LMR* lymphocyte to monocyte ratio, *NLR* neutrophil to lymphocyte ratio, *PLR* platelet lymphocyte ratio

Significant correlations were observed between the LMR and factors such as lymphocyte count (*p* < 0.0001), monocyte count (*p* < 0.0001), tumor size (*p* = 0.014), tumor depth (*p* = 0.0007), and TNM pStage (*p* = 0.0002). The NLR was significantly correlated with neutrophil count (*p* < 0.0001), lymphocyte count (*p* < 0.0001), and tumor depth (*p* = 0.002). Furthermore, significant correlations were observed between the PLR and lymphocyte count (*p* < 0.0001), platelet count (*p* < 0.0001), and tumor location (*p* = 0.042). It is notable that a low LMR was significantly correlated with more advanced TNM pStage, while the NLR and PLR showed no significant associations with TNM pStage.

### Prognostic factors for CSS in overall patients with esophageal cancer

Univariate analyses demonstrated that TNM pStage (*p* < 0.0001), tumor size (*p* = 0.0014), operation time (*p* = 0.0209), low LMR (*p* = 0.0008), and high PLR (*p* = 0.0232) were significant risk factors for poor prognosis (Table 2).

**Table 2:** Prognostic factors for cancer-specific survival in 147 patients with esophageal cancer

Variables	Patients (*n* = 147)	Category or characteristics	Univariate			Multivariate		
			HR	95 % CI	*p* value	HR	95 % CI	*p* value
Gender	15/132	(female/male)	0.942	0.406–2.740	0.9007			
Age	46/101	(70</≥70)	1.427	0.742–2.639	0.2771			
pStage	92/55	(1,2/3)	4.876	2.625–9.420	<0.0001	4.19	2.146–8.562	<0.0001
Tumor size	45/102	(3</≥3)	3.405	1.548–8.981	0.0014	1.433	0.580–4.056	0.4493
Operation time	99/48	(600</≥600)	2.041	1.116–3.741	0.0209	1.425	0.757–2.681	0.2699
Intraoperative blood loss	72/75	(500</≥500)	1.321	0.723–2.463	0.3663			
LMR	83/64	(≥4.0/4.0<)	2.829	1.537–5.378	0.0008	2.372	1.198–4.840	0.0129
NLR	37/110	(≥1.6/1.6<)	1.469	0.753–2.734	0.2494			
PLR	79/68	(147</≥147)	2.013	1.100–3.783	0.0232	1.12	0.611–2.404	0.5999
SCC antigen	109/38	(1.5</≥1.5)	1.3	0.603–2.564	0.4842			

*LMR* lymphocyte to monocyte ratio, *NLR* neutrophil to lymphocyte ratio, *PLR* platelet lymphocyte ratio, *SCC* squamous cell carcinoma, *HR* hazard ratio, *CI* confidence interval

TNM pStage (HR, 4.190; 95 % CI, 2.146–8.562; *p* < 0.0001) and low LMR (HR, 2.372; 95 % CI, 1.198–4.840; *p* = 0.0129) were found to be independently associated with poor prognosis via multivariate analysis (Table 2).

### Relationships between LMR, NLR, PLR, and clinicopathological features in non-elderly patients with esophageal cancer

The relationships between LMR, NLR, PLR, and clinicopathological features in non-elderly patients (younger than 70 years) are shown in Table 3. Significant correlations were observed between the LMR and such factors as lymphocyte count (*p* < 0.0001), monocyte count (*p* < 0.0001), tumor location (*p* = 0.0169), tumor size (*p* = 0.0309), tumor depth (*p* = 0.0093), and TNM pStage (*p* = 0.0003). The NLR was significantly correlated with neutrophil count (*p* < 0.0001), lymphocyte count (*p* < 0.0001), tumor size (*p* = 0.0452), tumor depth (*p* = 0.0018), and TNM pStage (*p* = 0.0032). Furthermore, significant correlations were observed between the PLR and lymphocyte count (*p* < 0.0001) as well as platelet count (*p* < 0.0001).

**Table 3:** Relationships between LMR, NLR, PLR and clinicopathologic features of 101 nonelderly patients

Characteristics	Total patients	LMR			NLR			PLR		
		<4 (*n* = 43)	≥4 (*n* = 58)	*p* value	1.6< (*n* = 25)	≥1.6 (*n* = 76)	*p* value	147< (*n* = 54)	≥147 (*n* = 47)	*p* value
Age (years)		61.9 ± 5.2	61.6 ± 5.6	0.7778	61.1 ± 5.8	61.9 ± 5.3	0.7249	62.4 ± 5.2	60.8 ± 5.5	0.1294
Gender				0.1283			0.2392			0.1171
Male	91	41	50		21	70		51	40	
Female	10	2	8		4	6		3	7	
WBC		6261.2 ± 2234.8	5951.4 ± 1747.8	0.7819	5654.4 ± 1725.4	6224.3 ± 2028.8	0.2101	6242.2 ± 1660.6	5900.6 ± 287.0	0.3863
Neutrophil		4020.2 ± 1757.4	3506.3 ± 1522.4	0.9402	2645.3 ± 978.7	4080.3 ± 1659.8	<0.0001	3481.2 ± 1252.0	4005.4 ± 1969.1	0.109
Lymphocyte		1352.8 ± 621.1	1964.2 ± 584.6	<0.0001	2362.7 ± 651.4	1487.2 ± 520.4	<0.0001	2068.7 ± 601.1	1284.7 ± 473.7	<0.0001
Monocyte		574.3 ± 223.8	336.1 ± 109.6	<0.0001	395.8 ± 163.2	451.3 ± 215.8	0.2417	438.1 ± 172.2	436.9 ± 238.6	0.9756
Platelet		230.1 ± 76.1	233.0 ± 70.2	0.8422	215.2 ± 64.4	237.2 ± 74.5	0.9051	205.7 ± 47.3	261.7 ± 84.3	<0.0001
Location of tumor				0.0169			0.5489			0.1445
Ce	4	4	0		0	4		0	4	
Ut	4	3	1		0	4		3	1	
Mt	49	23	26		14	35		24	25	
Lt	31	11	20		8	23		19	12	
Ae	13	2	11		3	10		8	5	
Tumor size (mm)		4.9 ± 2.1	3.9 ± 2.8	0.0309	3.4 ± 2.7	4.6 ± 2.5	0.0452	4.0 ± 2.8	4.7 ± 2.2	0.2116
Depth of tumor				0.0093			0.0018			0.0943
T1a-1b	44	12	32		13	31		29	15	
2	6	1	5		5	1		4	2	
3	40	23	17		5	35		17	23	
4a-4b	11	7	4		2	9		4	7	
Lymph node metastasis				0.5691			0.1307			0.3183
N0	56	22	34		18	38		32	24	
N1	28	13	15		6	22		16	12	
N2	6	4	2		1	5		3	3	
N3	11	4	7		0	11		3	8	
Pathological stage				0.0003			0.0032			0.1024
1a-1b	41	9	32		17	24		27	14	
2a-2b	20	15	5		1	19		8	12	
3a-3c	40	19	21		7	33		19	21	
Operation time (min)		617.8 ± 142.7	666.4 ± 148.0	0.101	643.33 ± 151.1	646.5 ± 146.8	0.9246	680.2 ± 147.9	606.0 ± 137.2	0.107
Intraoperative blood loss (ml)		727.9 ± 578.1	538.5 ± 523.1	0.0543	616.4 ± 567.6	620.1 ± 551.2	0.9772	563.0 ± 531.4	683.7 ± 574.5	0.2753
SCC antigen		1.01 ± 0.76	1.20 ± 1.26	0.3828	1.11 ± 1.26	1.11 ± 1.02	0.9667	1.04 ± 0.97	1.20 ± 1.19	0.465

*LMR* lymphocyte to monocyte ratio, *NLR* neutrophil to lymphocyte ratio, *PLR* platelet lymphocyte ratio

### Prognostic factors for CSS in non-elderly patients with esophageal cancer

In non-elderly patients, univariate analyses demonstrated that TNM pStage (*p* < 0.0001), tumor size (*p* = 0.0001), operation time (*p* = 0.0374), LMR (*p* < 0.0001), and PLR (*p* = 0.0189) were significantly associated with a poorer prognosis. Multivariate analysis demonstrated that TNM pStage (HR, 4.009; 95 % CI, 1.731–10.162; *p* = 0.001) and LMR (HR, 4.553; 95 % CI, 1.856–12.516; *p* = 0.0007) were independent risk factors for a poorer prognosis (Table 4).

**Table 4:** Univariate and multivariate analysis of prognostic factors in 101 non-elderly patients with esophageal cancer

Variables	Patients (*n* = 101)	Category or characteristics	Univariate			Multivariate		
			HR	95 % CI	*p* value	HR	95 % CI	*p* value
Gender	10/91	(female/male)	0.608	0.233–20.78	0.388			
pStage	61/40	(1,2/3)	5.022	2.321–11.715	<0.0001	4.009	1.731–10.162	0.001
Tumor size	34/67	(3</≥3)	8.34	2.491–51.782	0.0001	3.115	0.788–20.674	0.1114
Operation time	67/34	(600</≥600)	2.219	1.048–4.752	0.0374	1.109	0.490–2.540	0.803
Intraoperative blood loss	49/52	(500</≥500)	1.53	0.723–3.373	0.2679			
LMR	58/43	(≥4/4<)	5.076	2.259–12.909	<0.0001	4.553	1.856–12.516	0.0007
NLR	25/76	(≥1.6/1.6<)	1.593	0.656–4.750	0.322			
PLR	54/47	(147</≥147)	2.475	1.160–5.592	0.0189	1.163	0.499–2.845	0.5999
SCC antigen	76/25	(1.5</≥1.5)	0.915	0.305–2.244	0.857			

*LMR* lymphocyte to monocyte ratio, *NLR* neutrophil to lymphocyte ratio, *PLR* platelet lymphocyte ratio, *SCC* squamous cell carcinoma, *HR* hazard ratio, *CI* confidence interval

### Relationships between LMR, NLR, PLR, and clinicopathological features in elderly patients with esophageal cancer

The relationships between LMR, NLR, PLR, and clinicopathological features in elderly patients (70 years or older) are shown in Tables 5. Significant correlations were observed between the LMR and such factors as lymphocyte count (*p* < 0.0001), monocyte count (*p* = 0.0001), and serum SCC antigen (*p* = 0.0342). The NLR was significantly correlated with factors such as WBC (*p* = 0.0146), age (*p* = 0.012), lymphocyte count (*p* < 0.0001), and neutrophil count (*p* = 0.0009). Furthermore, significant correlations were observed between the PLR and lymphocyte count (*p* < 0.0001) as well as platelet count (*p* = 0.0009).

**Table 5:** Relationships between LMR, NLR, PLR and clinicopathologic features of 46 elderly patients

Characteristics	Total patients	LMR			NLR			PLR		
		<4 (*n* = 21)	≥4 (*n* = 25)	*p* value	1.6< (*n* = 12)	≥1.6 (*n* = 34)	*p* value	147< (*n* = 25)	≥147 (*n* = 21)	*p* value
Age (years)		74.0 ± 3.8	75.4 ± 4.4	0.8781	74.3 ± 3.0	75.0 ± 4.5	0.6094	76.2 ± 4.3	73.1 ± 3.3	0.012
Gender				0.2226			0.453			0.2226
Male	41	20	21		10	31		21	20	
Female	5	1	4		2	3		4	1	
WBC		5715.7 ± 1976.4	5596.0 ± 1891.6	0.835	4512.5 ± 1281.2	6052.4 ± 1946.8	0.0146	6080.0 ± 1881.6	5139.5 ± 1858.8	0.0966
Neutrophil		3790.0 ± 1932.6	3195.8 ± 1346.0	0.2271	2169.4 ± 828.4	3925.1 ± 1626.7	0.0009	3570.0 ± 1424.7	3344.5 ± 1909.4	0.649
Lymphocyte		1258.9 ± 352.0	1892.1 ± 593.0	<0.0001	1822.8 ± 528.1	1525.4 ± 594.0	0.1327	1943.9 ± 555.2	1197.2 ± 294.4	<0.0001
Monocyte		490.2 ± 174.3	311.6 ± 115.0	0.0001	344.1 ± 158.3	410.5 ± 171.8	0.2469	375.1 ± 164.4	414.7 ± 176.4	0.4351
Platelet		250.0 ± 85.7	212.8 ± 54.3	0.0805	263.8 ± 92.6	217.8 ± 60.4	0.0563	198.8 ± 53.8	266.6 ± 74.7	0.0009
Location of tumor				0.6568			0.1274			0.2753
Ce	2	1	1		1	1		0	2	
Ut	4	1	3		0	4		2	2	
Mt	16	6	10		6	10		8	8	
Lt	21	12	9		3	18		12	9	
Ae	3	1	2		2	1		3	0	
Tumor size (mm)		4.9 ± 1.5	3.9 ± 2.5	0.0987	4.6 ± 3.2	4.3 ± 1.7	0.6459	3.9 ± 1.8	4.9 ± 2.4	0.0987
Depth of tumor				0.0716			0.3997			0.2032
T1a-1b	22	8	14		5	17		11	11	
2	6	1	5		3	3		5	1	
3	16	10	6		3	13		9	7	
4a-4b	2	2	0		1	1		0	2	
Lymph node metastasis				0.1229			0.2441			0.0875
N0	23	8	15		4	19		11	12	
N1	14	6	8		6	8		9	5	
N2	6	4	2		2	4		5	1	
N3	3	3	0		0	3		0	3	
Pathological stage				0.0825			0.3939			0.8129
1a-1b	18	5	13		3	15		9	9	
2a-2b	13	6	7		5	8		8	5	
3a-3c	15	10	5		4	11		8	7	
Operation time (min)		700.0 ± 187.8	656.8 ± 185.7	0.4385	682.3 ± 227.8	674.5 ± 172.6	0.9021	668.5 ± 154.0	686.2 ± 221.6	0.7515
Intraoperative blood loss (ml)		800.7 ± 718.7	681.5 ± 840.3	0.3057	469.8 ± 368.8	829.9 ± 866.8	0.1723	675.2 ± 818.5	808.2 ± 746.9	0.2854
SCC antigen		1.56 ± 1.44	0.96 ± 0.68	0.0342	0.90 ± 0.80	1.35 ± 1.21	0.2379	1.07 ± 0.78	1.42 ± 1.43	0.2961

*LMR* lymphocyte to monocyte ratio, *NLR* neutrophil to lymphocyte ratio, *PLR* platelet lymphocyte ratio

### Prognostic factors for CSS in elderly patients with esophageal cancer

In elderly patients, univariate analysis demonstrated that that TNM pStage (*p* = 0.0023) was the only significant risk factor for a poor prognosis (Table 6).

**Table 6:** Univariate and multivariate analysis of prognostic factors in 46 elderly patients with esophageal cancer

Variables	Patients (*n* = 46)	Category or characteristics	Univariate			Multivariate		
			HR	95 % CI	*p* value	HR	95 % CI	*p* value
Gender	5/41	(female/male)	3.114	0.611–56.892	0.201			
pStage	31/15	(1,2/3)	5.22	1.824–16.080	0.0023	5.22	1.824–16.080	0.0023
Tumor size	11/35	(3</≥3)	0.976	0.333–3.529	0.9666			
Operation time	32/14	(600</≥600)	1.761	0.615–4.929	0.2822			
Intraoperative blood loss	23/23	(500</≥500)	0.981	0.349–2.820	0.9707			
LMR	25/21	(≥4/4<)	1.118	0.368–3.175	0.837			
NLR	12/34	(≥1.6/1.6<)	0.853	0.464–1.535	0.718			
PLR	25/21	(147</≥147)	1.3	0.464–3.712	0.616			
SCC antigen	33/13	(1.5</≥1.5)	2.261	0.689–6.565	0.167			

*LMR* lymphocyte to monocyte ratio, *NLR* neutrophil to lymphocyte ratio, *PLR* platelet lymphocyte ratio, *SCC* squamous cell carcinoma, *HR* hazard ratio, *CI*, confidence interval

### Postoperative CSS based on LMR, NLR, and PLR in all patients with esophageal cancer

**Fig. 1:**
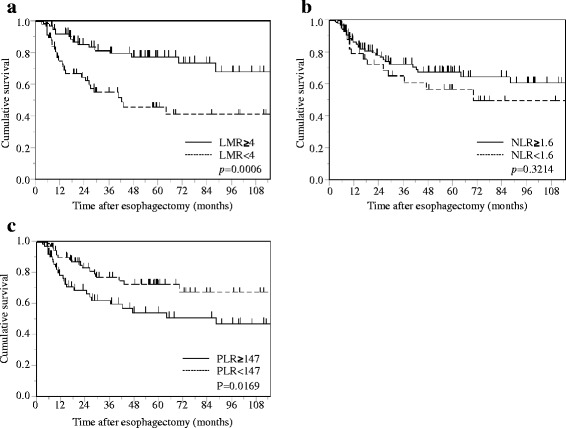
Kaplan-Meier survival curves showing CSS after curative esophagectomy in overall patients with esophageal cancer. **a** LMR. **b** NLR. **c** PLR

Patients with a low LMR had a significantly poorer prognosis in terms of CSS than those with a high LMR (*p* = 0.0006). In contrast, patients with a high PLR had a significantly poorer prognosis than those with a low PLR (*p* = 0.0169), whereas no significant differences in CSS were observed between patients with a low or high NLR (*p* = 0.3214; Fig. 1a-c).

### Postoperative CSS based on LMR, NLR, and PLR in non-elderly patients with esophageal cancer

**Fig. 2:**
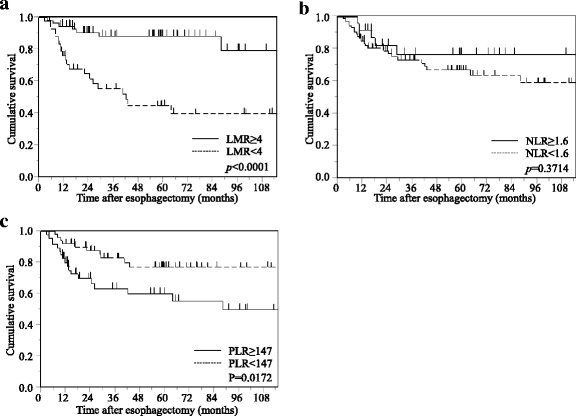
Kaplan-Meier survival curves showing CSS after curative esophagectomy in non-elderly patients with esophageal cancer. **a** LMR. **b** NLR. **c** PLR

Patients with a low LMR had a significantly poorer prognosis in terms of CSS than those with a high LMR (*p* < 0.0001). In contrast, patients with a high PLR had a significantly poorer prognosis than those with a low PLR (*p* = 0.0172), whereas no significant differences in CSS were observed between patients with a low or high NLR (*p* = 0.3714; Fig. 2a-c).

### Postoperative CSS based on LMR, NLR, and PLR in elderly patients with esophageal cancer

**Fig. 3:**
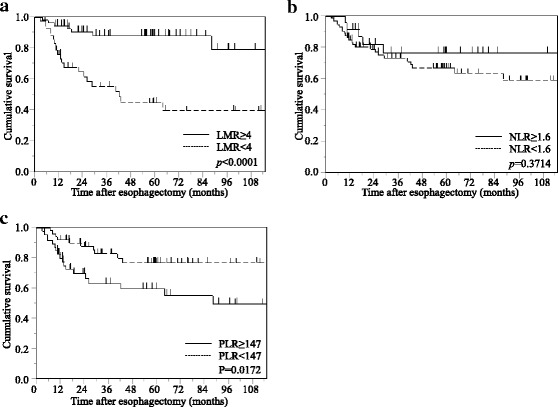
Kaplan-Meier survival curves showing CSS after curative esophagectomy in elderly patients with esophageal cancer. **a** LMR. **b** NLR. **c** PLR

In the elderly group, no significant differences in CSS were observed between patients with either low or high LMR (*p* = 0.4700), NLR (*p* = 0.9698), or PLR (*p* = 0.5386; Fig. 3a-c).

## Discussion

Pathological features, including tumor stage, nodal status, and resection margin, are considered important in determining cancer patient survival [11]. However, it is now clear that cancer survival is not solely determined by tumor pathology; indeed, recent studies have shown that preoperative inflammation-based prognostic scores can predict the overall survival of patients with various types of cancers [2–4]. In the present study, we retrospectively analyzed the clinical data of patients undergoing a potentially curative resection for esophageal cancer to determine whether the LMR, NLR, and PLR have prognostic values according to each TNM pStage. The results demonstrated that the LMR can be used as a novel predictor of postoperative CSS in patients with esophageal cancer after curative esophagectomy. Additionally, univariate analyses revealed that a low LMR was a significant risk factor for poor prognosis in stage III patients, whereas no prognostic factor was detected in patients with stage I or II cancer.

Interleukin-6 (IL-6) is a multifunctional inflammatory cytokine that triggers the proliferation and differentiation of a variety of cell types, including immune competent cells and hematopoietic cells. IL-6 induces not only neutrophil proliferation, but also the differentiation of megakaryocytes to platelets, and these events are similar to those underlying the systemic inflammatory response (SIR) [12, 13]. Theoretically, dynamic changes in the SIR resulting from tumor-host interactions are best estimated by directly measuring the serum IL-6 level. However, routine measurement of IL-6 in cancer patients in the clinical setting is expensive and inconvenient. On the other hand, the LMR, NLR, and PLR are based on blood cell components whose levels are regulated by cytokines, most notably, IL-6; these blood cell components proliferate and differentiate immediately after inflammatory cytokine release [14]. Moreover, measurement of the LMR, NLR, and PLR is easy, convenient, and cost-effective and therefore can be performed routinely.

In this study, we examined the prognostic significance of the LMR, NLR, and PLR in both elderly and non-elderly patients undergoing thoracoscopic esophagectomy for esophageal cancer. Esophageal cancer is the eighth most common cancer and the sixth most common cause of cancer deaths worldwide [15]. It occurs predominantly in elderly people, and the average age at the time of diagnosis continues to rise, with a peak incidence between 70 and 75 years of age [16]. Because age-specific prognostic factors in patients with esophageal cancer have not yet been described, we divided patients into two age groups in order to determine the age-specific prognostic values of the LMR, NLR, and PLR. The reason we chose a cut-off value of 70 years is because “elderly” is typically defined as a patient aged over 70 years in a plurality of studies on elderly patients with esophageal cancer [17–19].

Platelets are a key element linking the processes of hemostasis, inflammation, and tissue repair. Previous studies have shown that proinflammatory mediators stimulate megakaryocyte proliferation and are responsible for platelet production [20, 21]. Consequently, platelet activation causes angiogenic growth factor release as well as platelet adherence to tumor microvessels and extravasation via increased vascular permeability; this process leads to platelet activation [22, 23]. Lymphocytes can cause systemic inflammation by releasing numerous inhibitory immunologic mediators, particularly interleukin-10 and transforming growth factor-ß, which may consequently cause suppression of antitumor immunity via decreased regulatory T cell levels [6]. Accordingly, there is increasing evidence that lymphocytes are essential for antitumor immune reactions owing to several mechanisms, including the ability to enhance tumor cell apoptosis, inhibition of tumor cell proliferation, and promotion of metastasis [24]. Neutrophils are known to not only produce angiogenic cytokines, but have also been shown to generate matrix metalloproteinase-9, which induces an angiogenic state in cancer cells [25].

Based on such inflammatory responses, systemic inflammatory markers such as the LMR, NLR, and PLR have been shown to predict mortality and recurrence in a variety of cancers, but their role in esophageal cancer remains controversial [7, 20, 26].

We revealed that a low LMR in patients with esophageal cancer was significantly correlated with more advanced TNM pStage (*p* = 0.0002), but a low LMR was found to be independently associated with poor prognosis via multivariate analysis (HR, 2.372; *p* = 0.0129), as determined by Kaplan-Meier analysis and a log-rank test (*p* = 0.0006). A definitive explanation for our findings remains speculative. Monocytes are known to promote tumorigenesis and angiogenesis through local immune suppression and stimulation of tumor neovasculogenesis [25]. Moreover, tumor-associated macrophages developing from mononuclear cell lineages have been demonstrated to be able to inhibit cancer progression and spread of metastatic tumors [27, 28]. This could explain why an elevated monocyte count confers poor clinical outcomes in various types of cancers [29]. A poor prognosis was observed in patients with a low LMR in this study, which is reasonable because both lymphopenia and monocytosis induce immune suppression, as mentioned above. Moreover, the results of subgroup analysis revealed that the preoperative LMR was the most significant prognostic factor in non-elderly patients (HR, 4.553; *p* = 0.0007), as determined by Kaplan-Meier analysis and a log-rank test (*p* < 0.0001), but not in elderly patients. The present study may have failed to demonstrate a prognostic significance of the LMR in elderly patients because these patients were more likely to have advanced age-related conditions that cause immune suppression. Further investigations are required to elucidate the precise mechanisms that affect the prognosis of esophageal cancer patients.

Changes in platelet count and platelet function have been identified as part of a paraneoplastic syndrome in many cancers [30], and a high platelet count was found to be closely associated with TNM pStage, metastasis, as well as a high risk of recurrence in many types of cancer [31, 32]. Consequently, the PLR may act as a marker of the balance between host inflammatory and immune responses. However, to the best of our knowledge, the relationship between the PLR and esophageal cancer has not yet been described. We therefore focused on the PLR and CSS in esophageal cancer patients. Although univariate analysis demonstrated that the PLR was a significant risk factor for poorer CSS, as determined by Kaplan-Meier analysis and a log-rank test (*p* = 0.0169), multivariate analysis failed to confirm that the PLR was a significant predictor of CSS. Similarly, in non-elderly patients, univariate analysis demonstrated that the PLR was a significant risk factor for poorer CSS (*p* = 0.0172), but this significance was lost when analysis was confined to elderly patients. Recent studies have demonstrated that termed combination of platelet count and mean platelet volume is a predictor for postoperative survival in esophageal cancer patients [33]. Further studies are necessary to examine the role of these inflammatory biomarkers in various types of cancers.

The NLR has been reported to be highly promising in stratifying the outcome in large cohorts of patients with cancer [34, 35]. The relationship between the NLR and prognosis is probably complex and remains unclear. Recently, many studies have shown that a high NLR may indicate an impaired host immune response to the tumor [36]. In this study, the NLR did not affect the prognosis of esophageal cancer patients following curative resection, which may be due to the small retrospective sample size and short follow-up duration of the study. However, other components of the systemic inflammatory response, including cytokines and chemokines, have proven prognostically important in some studies [37].

There were several potential limitations that warrant consideration in our study, which include single-institution retrospective analysis, short follow-up periods, and a small sample size, especially elderly patients. Furthermore, we excluded patients who had received adjuvant chemotherapy and/or radiotherapy, which may have influenced our analysis. Thus, large, prospective, randomized controlled trials are needed to confirm these preliminary results. In addition, the amount of weight loss are well-known prognostic factors for various types of cancers. Minimal weight loss and a good performance status are considered favorable prognostic factors. Needless to say significant weight loss may impact bone marrow function as well as the patient’s ability to mount a host-tumor response. But we could not reveal that weight loss were proven to be independent prognostic factors in esophageal cancer, because our study is retrospective analysis, and data about the weight loss are insufficient.

## Conclusion

In conclusion, our study demonstrated that the LMR and PLR were associated with CSS of esophageal cancer patients after curative esophagectomy. Moreover, the results of subgroup analysis revealed that the preoperative LMR and PLR were the most significant prognostic factors in non-elderly patients, as determined by Kaplan-Meier analyses and log-rank tests. In particular, a low LMR was a significant and independent predictor of poor survival. In non-elderly patients, a low LMR was also an independent risk factor for a poorer prognosis. The LMR and PLR are convenient, cost effective, and readily available as a part of routine complete blood counts, and could thus act as markers of survival in this malignancy.

## Abbreviations

AUC: Area under curve
CBC: Complete blood cell
CSS: Cancer-specific survival
LMR: Lymphocyte to monocyte ratio
NLR: Neutrophil to lymphocyte ratio
PLR: Platelet lymphocyte ratio
ROC: Receiver operating curve
SCC: Squamous cell carcinoma
